# Cytogenetic analysis of *Hypomasticus copelandii* and *H. steindachneri*: relevance of cytotaxonomic markers in the Anostomidae family (Characiformes)

**DOI:** 10.3897/compcytogen.v15.i1.61957

**Published:** 2021-03-10

**Authors:** Filipe Schitini Salgado, Marina Souza Cunha, Silvana Melo, Jorge Abdala Dergam

**Affiliations:** 1 Departamento de Biologia Animal, Universidade Federal de Viçosa, Av. P. H. Rolfs, s/n, Centro, Viçosa, 36570-900, Minas Gerais, Brazil; 2 Departamento de Biologia Geral, Universidade Federal de Viçosa, Av. P. H. Rolfs, s/n, Centro, Viçosa, 36570-900, Minas Gerais, Brazil; 3 Departamento de Morfologia, Instituto de Biociências, Universidade Estadual Paulista, R. Prof. Dr. Antônio Celso Wagner Zanin, s/n, Distrito de Rubião Junior, Botucatu, 18618-689, São Paulo, Brazil

**Keywords:** Anastomid, coastal basins, cytogenetics, endemic species, fluorescence *in situ* hybridization, freshwater fishes, repetitive sequences

## Abstract

Recent phylogenetic hypotheses within Anostomidae, based on morphological and molecular data, resulted in the description of new genera (*Megaleporinus* Ramirez, Birindelli et Galetti, 2017) and the synonymization of others, such as the reallocation of *Leporinus copelandii* Steindachner, 1875 and *Leporinus steindachneri* Eigenmann, 1907 to *Hypomasticus* Borodin, 1929. Despite high levels of conservatism of the chromosomal macrostructure in this family, species groups have been corroborated using banding patterns and the presence of different sex chromosome systems. Due to the absence of cytogenetic studies in *H. copelandii* (Steindachner, 1875) and *H. steindachneri* (Eigenmann, 1907), the goal of this study was to characterize their karyotypes and investigate the presence/absence of sex chromosome systems using different repetitive DNA probes. Cytogenetic techniques included: Giemsa staining, Ag-NOR banding and FISH using 18S and 5S rDNA probes, as well as microsatellite probes (CA)_15_ and (GA)_15_. Both species had 2n = 54, absence of heteromorphic sex chromosomes, one chromosome pair bearing Ag-NOR, 18S and 5S rDNA regions. The (CA)_15_ and (GA)_15_ probes marked mainly the subtelomeric regions of all chromosomes and were useful as species-specific chromosomal markers. Our results underline that chromosomal macrostructure is congruent with higher systematic arrangements in Anostomidae, while microsatellite probes are informative about autapomorphic differences between species.

## Introduction

Within the order Characiformes, the family Anostomidae encompasses around 150 valid species distributed throughout South America (Froese and Pauly 2019; Fricke et al. 2020). Fish of this family carry out annual reproductive migrations and constitute a large part of the fish biomass in several aquatic habitats, representing an important resource for human activities (Garavello and Britski 2003). Up to now, seven anostomid species are considered endangered and many others need urgent assessment of their conservational status (reviewed in Birindelli et al. 2020). In many cases, original type series are composed of more than one species, such as the case of *Leporinus copelandii* Steindachner, 1875 (Birindelli et al. 2020).

Recently, phylogenetic hypotheses based on morphological and molecular data have suggested the creation of the new genus *Megaleporinus* Ramirez, Birindelli et Galetti, 2017 (Ramirez et al. 2016, 2017), and the synonymization of others, such as the reallocation of *L. copelandii* and *Leporinus steindachneri* Eigenmann, 1907 to *Hypomasticus* Borodin, 1929 (Birindelli et al. 2020). Even with these proposed changes, both *Leporinus* Agassiz, 1829 and *Hypomasticus* are still not monophyletic, requiring further taxonomic investigations.

Cytogenetic studies in this group have revealed a conserved karyotype macrostructure of 2n = 54 and fundamental number (NF) = 108 (Table 1). Regardless of this conservatism, the cytogenetic banding patterns, the differential accumulation of repetitive DNA, and the presence/absence of sex chromosome systems have been useful to help species identification in this family (reviewed in Barros et al. 2017). Both *Hypomasticus copelandii* (Steindachner, 1875) and *Hypomasticus steindachneri* (Eigenmann, 1907) had an early divergence in the phylogeny of the family (Ramirez et al. 2016, 2017; Birindelli et al. 2020), and were never analyzed cytogenetically. Therefore, the goal of this paper was to characterize their karyotypes and to investigate the presence/absence of sex chromosome systems using different repetitive DNA probes in these two species from Brazilian southeastern coastal basins in order to identify potential cytotaxonomic markers. We also provided a review of the cytogenetic data available for the family Anostomidae.

**Table 1.** Cytogenetic data available on the Anostomidae species regarding their chromosome number (2*n*), karyotype description, presence or absence of sex-chromosome systems, number of chromosomes marked by the Ag-NOR banding technique, and also 18S and 5S rDNA probes.

Species	2*n*	Karyotype	Sex-System	Ag-NOR	18S	5S	References
*Abramites hypselonotus*	54	–	no	–	2	–	Silva et al. 2013
*A. solaria*	54	–	no	2	–	–	Martins et al. 2000
*Anostomus ternetzi*	54	–	no	2	–	–	Martins et al. 2000
*Hypomasticus copelandii*	54	28m+26sm	no	2	2	2	**Present Study**
*H. steindachneri*	54	30m+24sm	no	2	2	2	**Present Study**
*Laemolyta taeniata*	54	28m+26sm	no	2	2	2 †	Barros et al. 2017
*Leporellus vittatus*	54	28m+26sm	no	2	2	2–4 †	Galetti Jr et al. 1984; Dulz et al. 2019
*Leporinus agassizi*	54	28m+26sm	no	2	2	2	Barros et al. 2017
*L. amblyrhyncus*	54	–	no	2	–	–	Galetti Jr et al. 1991
*L. fasciatus*	54	28m+26sm	no	2	2	2	Barros et al. 2017
*L. friderici*	54	28m+26sm/32m+22sm	no	2	2	2–4	Martins and Galetti Jr., 1999; Silva et al. 2012; Borba et al. 2013; Barros et al. 2017; Ponzio et al. 2018; Dulz et al. 2019; Crepaldi and Parise-Maltempi 2020
*L. lacustris*	54	30m+24sm	no	2	2	–	Galetti Jr et al. 1981; Galetti Jr et al. 1984; Mestriner et al. 1995; Silva et al. 2012, 2013; Borba et al. 2013
*L. multimaculatus*	54	26m+28sm	ZZ/ZW	2	–	–	Barros et al. 2018; Venere et al. 2004
*L. octofasciatus*	54	–	no	2	–	–	Galetti Jr et al. 1984
*L. piau*	54	–	no	2	–	–	Galetti Jr et al. 1991
*L. striatus*	54	–	no	2	2	–	Galetti Jr et al. 1991; Silva et al. 2012, 2013; Borba et al. 2013; Ponzio et al. 2018
*L. taeniatus*	54	–	no	2	–	–	Galetti Jr et al. 1991
*Megaleporinus conirostris* ‡	54	–	ZZ/ZW	2	–	–	Galetti Jr et al. 1995
*M. elongatus* ‡	54		Z_1_Z_1_Z_2_Z_2_/Z_1_W_1_Z_2_W_2_	2	2	4	Martins and Galetti Jr. 2000; Parise-Maltempi et al. 2007, 2013; Marreta et al. 2012; Silva et al. 2012, 2013; Borba et al. 2013; Ponzio et al. 2018; Crepaldi and Parise-Maltempi 2020
*M. macrocephalus* ‡	54	–	ZZ/ZW	–	2	–	Galetti Jr and Foresti 1986; Galetti Jr et al. 1995; Silva et al. 2012, 2013; Borba et al. 2013; Ponzio et al. 2018; Utsunomia et al. 2019; Crepaldi and Parise-Maltempi 2020
*M. obtusidens* ‡	54	26m+28sm/ 28m+26sm	ZZ/ZW	2	2	2–4	Galetti Jr et al. 1981; Galetti Jr et al. 1995; Martins and Galetti Jr. 2000; Silva et al. 2012, 2013; Borba et al. 2013; Utsunomia et al. 2019; Dulz et al. 2020
*M. reinhardti* ‡	54	28m+26sm	ZZ/ZW	–	2	2	Galetti Jr and Foresti 1986; Galetti Jr et al. 1995; Dulz et al. 2020
*M. trifasciatus* ‡	54	26m+28sm	ZZ/ZW	2–3	6 §	2 †	Galetti Jr et al. 1995; Barros et al. 2017
*Pseudanos trimaculatus*	54	–	no	2	–	–	Martins et al. 2000
*Rhytiodus microlepis*	54	28m+26sm	no	2	4 §	2	Barros et al. 2017
*Schizodon altoparanae*	54	–	no	2	–	4	Martins and Galetti Jr. 2000
*S. borellii*	54	–	no	2	2	4	Martins and Galetti Jr. 2000; Silva et al. 2012, 2013; Ponzio et al. 2018
*S. fasciatus*	54	28m+26sm	no	2	22 §	2 †	Barros et al. 2017
*S. intermedius*	54	–	no	2	–	–	Martins and Galetti Jr. 1997
*S. isognathus*	54	–	no	2	2	4	Martins and Galetti Jr. 2000; Silva et al. 2012, 2013; Ponzio et al. 2018
*S. knerii*	54	–	no	2	–	4	Martins and Galetti Jr. 2000
*S. nasutus*	54	–	no	2	–	4	Martins and Galetti Jr. 2000
*S. vittatus*	54	–	no	2	–	4	Martins and Galetti Jr. 2000

† indicates synteny between 18S and 5S rDNA clusters. ‡ Species were assigned to the new genus *Megaleporinus* according to Ramirez et al. (2017). § Barros et al. (2017) did not exclude the possibility of technical artifacts and suggested that the expansion of the rDNA sites should be confirmed with supplementary analysis.

## Material and methods

### Sample collection

*Hypomasticus copelandii* was collected from Glória (Paraíba do Sul River Basin), Itabapoana (Itabapoana River Basin), Matipó (Doce River Basin) and Mucuri (Mucuri River Basin) rivers, covering its full range of distribution in southeastern Brazil. *Hypomasticus steindachneri* was collected from Tiririca Lake (Doce River Basin) (Table 2). Collection permit of the Instituto Chico Mendes de Biodiversidade (ICMBio) (SISBIO14975-1) was issued to Jorge Abdala Dergam. Species identification followed Garavello (1979) and the sex identification was made through histological analysis. Voucher specimens were deposited in the scientific collection of the Museu de Zoologia João Moojen in Viçosa, Minas Gerais, Brazil (Table 2).

**Table 2.** Locales and sample size of *Hypomasticus copelandii* and *Hypomasticus steindachneri* from southeastern Brazil.

Species	Voucher	Locality	GPS coordinates	Sample size
				Male/Female
*Hypomasticus copelandii*	MZUFV4500 MZUFV 4504	Glória River, Paraíba do Sul River Basin	21°05'21"S, 42°20'30"W	01/02
	MZUFV4503 MZUFV 4504	Itabapoana River, Itabapoana River Basin	20°59'26"S, 41°42'56"W	02/02
	MZUFV4502	Matipó River, Doce River Basin	20°06'59"S, 42°24'14"W	04/04
	MZUFV4354	Mucuri River, Mucuri River Basin	17°42'21"S, 40°45'42"W	0/1
*Hypomasticus steindachneri*	MZUFV3596 MZUFV3607 MZUFV3635 MZUFV4658	Tiririca Lake, Doce River Basin	19°18'51"S, 42°24'13"W	4/4

### Cytogenetic analyses

The specimens were anesthetized with clove oil 300 mg.L^-1^ (Lucena et al. 2013) as approved by the Universidade Federal de Viçosa Animal Welfare Committee (CEUA authorization 08/2016). Mitotic chromosomes were obtained from a direct method using kidney (Bertollo et al. 1978) and the following cytogenetic techniques were used: conventional staining with Giemsa 5% diluted in Sorensen buffer (0.06M, pH 6.8) for basic karyotypic analysis, identification of the argyrophilic nucleolar organizer regions through Ag-NOR banding technique (Howell and Black 1980), and fluorescence *in situ* hybridization (FISH) following the protocol outlined in Pinkel et al. (1986) using 18S and 5S rDNA probes, as well as (CA)_15_ and (GA)_15_ microsatellite probes. The ribosomal probes were obtained through polymerase chain reaction (PCR) using the following primers: 18Sf (5'-CCG CTT TGG TGA CTC TTG AT-3') and 18Sr (5'-CCG AGG ACC TCA CTA AAC CA-3') (Gross et al. 2010); 5Sa (5'-TAC GCC CGA TCT CGT CCG ATC-3') and 5Sb (5'-CAG GCT GGT ATG GCC GTA AGC-3') (Martins et al. 2006). The ribosomal genes were labeled with digoxigenin-11-dUTP (Roche Applied Science) and the signal was detected with anti-digoxigenin-rhodamine (Roche Applied Science), whereas the microsatellite probes were synthesized and labeled with Cy3 fluorochrome at the 5' end (Sigma).

Digital images were captured in a BX53F Olympus microscope equipped with DP73 and MX10 Olympus camera for classical and molecular techniques respectively, both using the CellSens imaging software. Chromosomes were measured with the Image-Pro Plus software and classified according to their size and arm ratios as metacentric (m) or submetacentric (sm) (Levan et al. 1964). At least five metaphases from each individual were analyzed in order to determine the chromosomal patterns.

## Results

**Figure 1.**
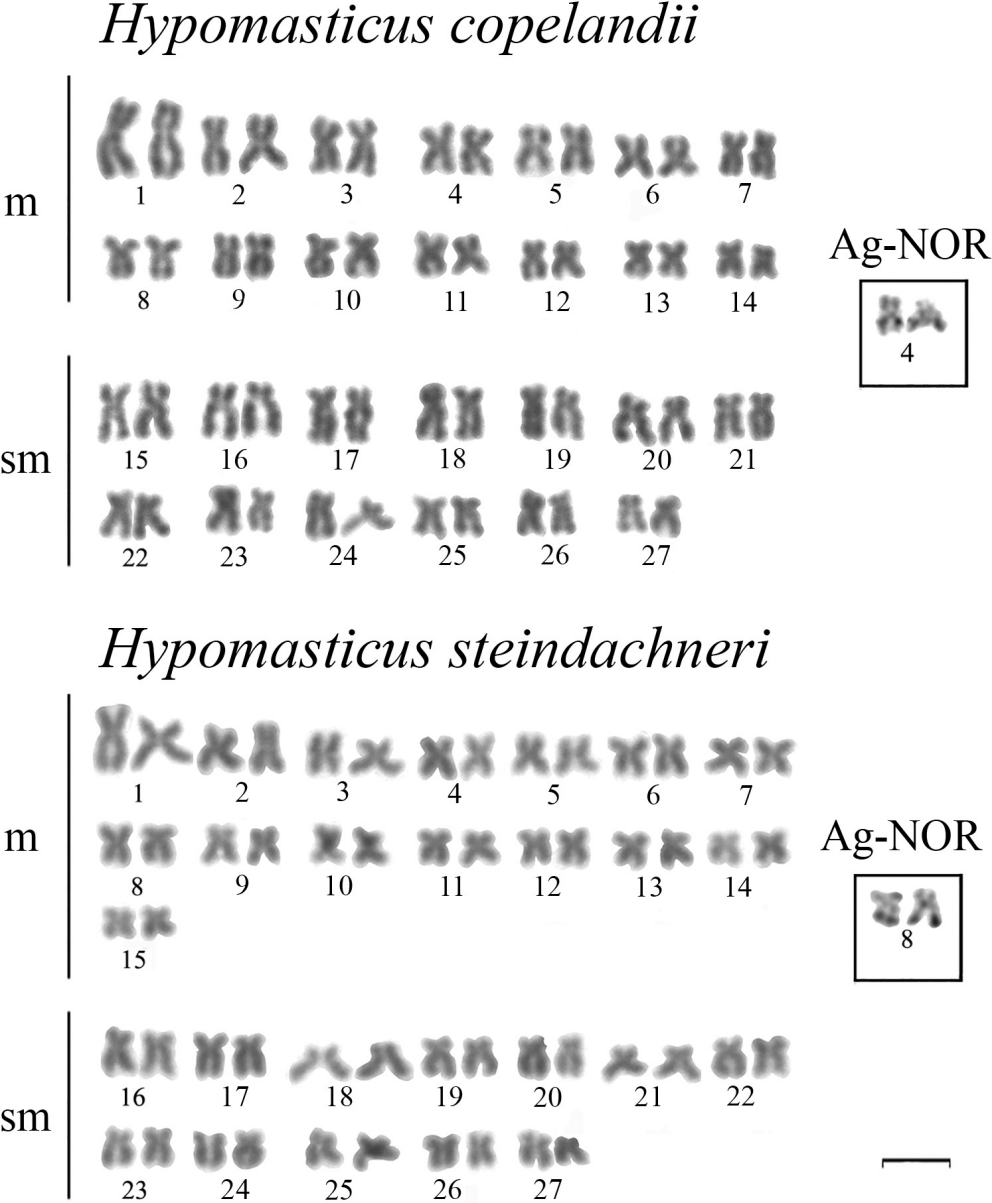
Giemsa-stained karyotypes of *Hypomasticus copelandii* and *Hypomasticus steindachneri*. Ag-NORs are shown in the boxes. Scale bar: 10 μm.

**Figure 2.**
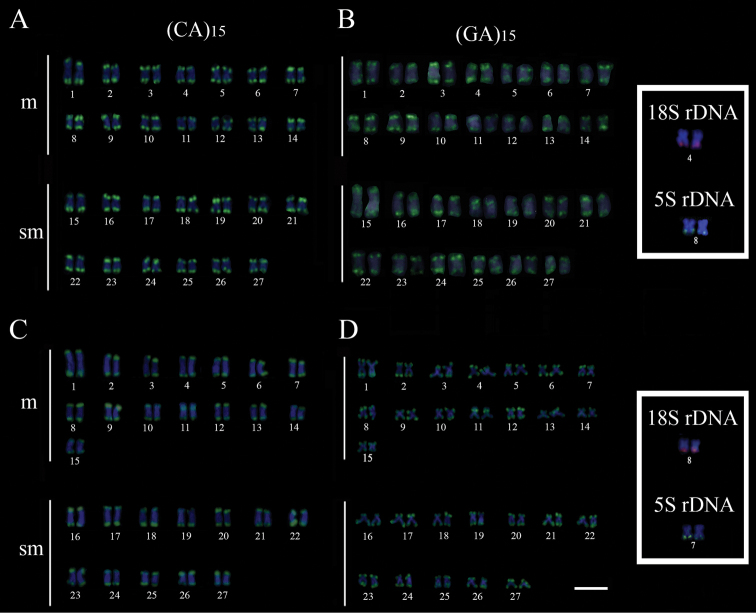
Cytogenetic FISH patterns on *Hypomasticus copelandii* (**A, B**) and *Hypomasticus steindachneri* (**C, D**). Left column (CA)_15_ probe (**A–C**). Right column (GA)_15_ probe (**B–D**). 18S and 5S rDNA probes are shown in the boxes. Scale bar: 5 μm.

Our results showed 2n = 54 in all *H. copelandii* populations, karyotype of 28m + 26sm and NF = 108, no heteromorphic sex chromosomes were detected, and Ag-NOR was located at the terminal region of chromosome pair 4 (Fig. 1). *H. steindachneri* showed 2n = 54, karyotype of 30m + 24sm and NF = 108, also without heteromorphic sex chromosomes, and Ag-NOR was located at the terminal region of chromosome pair 8 (boxes in Fig. 1). The 18S rDNA signals were detected at the terminal region of chromosome pair 4 in *H. copelandii* and pair 8 in *H. steindachneri*, whereas the 5S rDNA signals were detected at the interstitial region of chromosome pair 8 in *H. copelandii* and pair 7 in *H. steindachneri* (boxes in Fig. 2).

The microsatellite (CA)_15_ was detected in both arms of all chromosomes in *H. copelandii*, whereas microsatellite (GA)_15_ showed the same pattern with the exception of submetacentric pair 18 that showed signals in the interstitial region of the short arm (Fig. 2). Probes (CA)_15_ and (GA)_15_ exhibited the same general pattern in *H. steindachneri*, terminal markings in both arms of all chromosomes, except for metacentric pair 11, which showed interstitial signals in the short arm with both probes (Fig. 2). These distinctive markings obtained with the microsatellites were consistently observed in both sexes.

## Discussion

**Figure 3.**
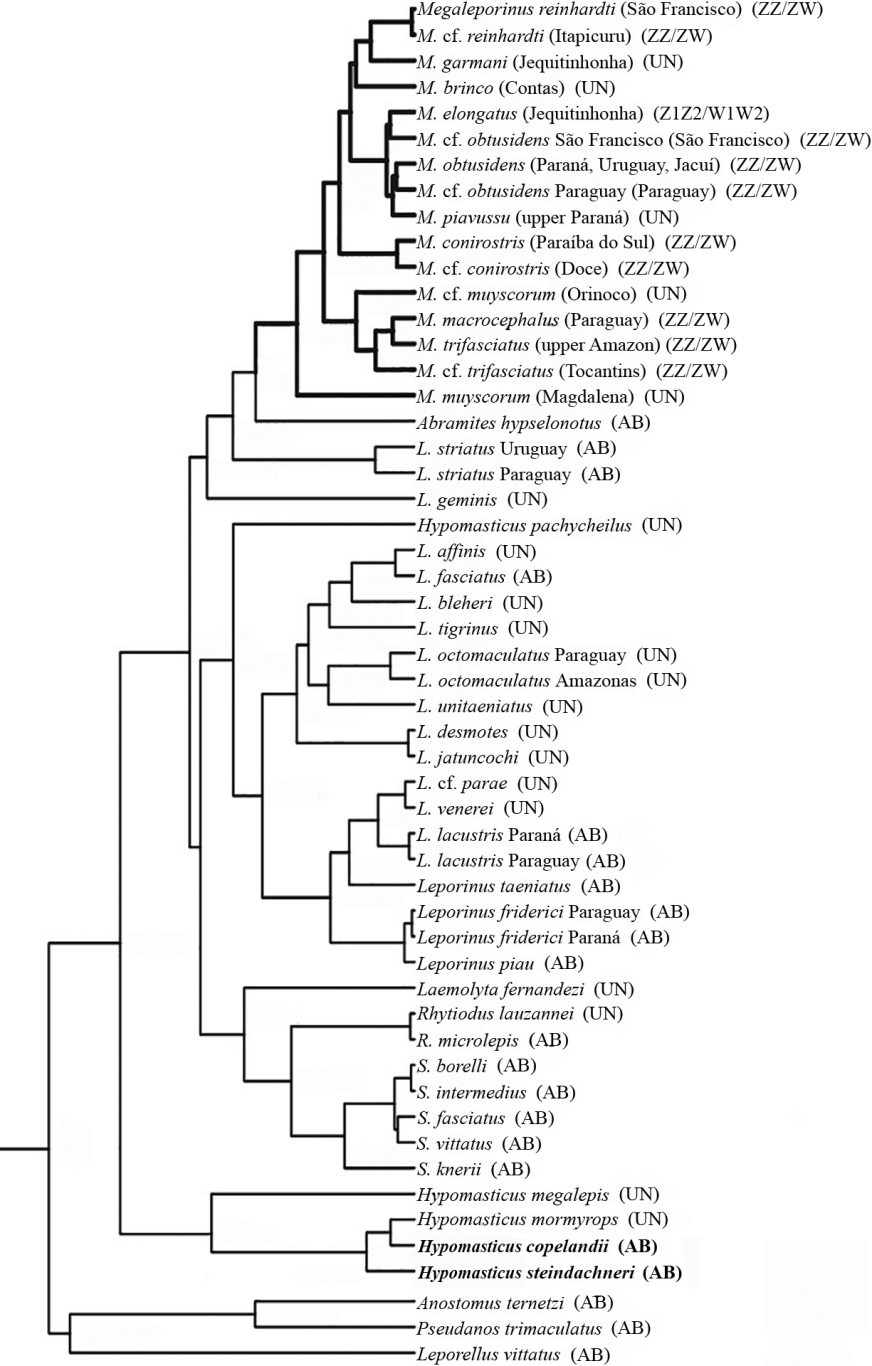
Phylogenetic tree of the Anostomidae family adapted from Ramirez et al. (2017) and Birindelli et al. (2020) including all cytogenetic information available regarding presence or absence of sex chromosome systems. AB: Absent; UN: Unknown.

The conserved Anostomidae karyotype macrostructure is observed in both *H. copelandii* and *H. steindachneri*, i.e. 2*n* = 54 and NF = 108, with some differences in the karyotypic formula regarding the number of metacentric and submetacentric chromosomes (Table 1). The absence of heteromorphic sex chromosomes reflects their early divergence in the phylogeny of the family (Ramirez et al. 2016, 2017; Birindelli et al. 2020). This is the first cytogenetic report for the genus *Hypomasticus* indicating that the absence of a sex chromosome system constitutes a plesiomorphic trait within Anostomidae (Fig. 3).

Ramirez et al. (2017) proposed the creation of *Megaleporinus* based on morphological, molecular and cytogenetic data, synonymizing some *Leporinus* and *Hypomasticus* species, and considering the ZZ/ZW sex system as a synapomorphic trait of this new genus. This hypothesis has been corroborated by other studies, which also included *Megaleporinus elongatus* (Valenciennes, 1850) with a Z_1_Z_2_/W_1_W_2_ multiple sex chromosome system (Parise-Maltempi et al. 2007, 2013; Marreta et al. 2012; Barros et al. 2018; Crepaldi and Parise-Maltempi 2020). However, not all current *Megaleporinus* species have been karyotyped (Fig. 3), and a ZZ/ZW system has also been observed in *Leporinus multimaculatus* Birindelli, Teixeira et Britski, 2016, which may have arisen independently (Venere et al. 2004; Barros et al. 2018). The inclusion of this species in the phylogenetic analyzes will help to elucidate this question, as well as the cytogenetic characterization of the remaining *Megaleporinus* spp.

Although Ag-NOR number is conserved for most anastomid species with only two markings (Table 1), the chromosome locus characterizes each species, comprising a species-specific character useful as an efficient cytotaxonomic marker (Galetti Jr et al. 1984, 1991; Barros et al. 2017). High correlation between Ag-NOR banding and 18S rDNA FISH technique is also a conserved pattern in the family, with only three exceptions (Table 1). Barros et al. (2017) acknowledged that this discrepancy observed on these three species could be due to technical artifacts and suggested that the expansion of the 18S rDNA sites in Anostomidae should be verified with supplementary analysis. The 18S and 5S rDNA probes were not co-located in neither *H. copelandii* nor *H. steindachneri*, as observed in most species of the family (Table 1), although it remains to be confirmed with double-FISH analysis, as syntenic sites have been observed in other species of the family, such as in *Megaleporinus trifasciatus* (Steindachner, 1876), *Laemolyta taeniata* (Kner, 1858), *Schizodon fasciatus* Spix et Agassiz, 1829 (Barros et al. 2017), and *Leporellus vittatus* (Valenciennes, 1850) (Dulz et al. 2019).

In Anostomidae, 5S rDNA variation is restricted to two or four markings and, interestingly, with intraspecific variation among populations in a few species (Table 1). These intraspecific variations call attention to the importance of populational studies to highlight species genetic diversity, important to delineate conservational strategies (Paiva et al. 2006; Abdul-Muneer 2014). Specially in the cases of migratory species, where the highly fragmented habitats could cause isolation of gene flow (Santos et al. 2013). The identical cytogenetic patterns observed in all *H. copelandii* populations, covering its full distribution range, indicate absence of genetic structure.

Microsatellite (CA)_15_ and (GA)_15_ probes marked the terminal region of both arms in most of the chromosomes in both species, a pattern that is observed in the autosomes of species with sex chromosome systems, whereas the heteromorphic sex chromosomes have specific accumulation patterns of distinct repetitive DNA classes (Parise-Maltempi et al. 2007; Cioffi et al. 2012; Marreta et al. 2012; Poltronieri et al. 2014; Utsunomia et al. 2019; Dulz et al. 2020). The differential interstitial markings, observed in both male and female chromosome complements, can be used as an additional cytotaxonomic marker to distinguish *H. copelandii* from *H. steindachneri* (Fig. 2), and also from species with heteromorphic sex chromosomes (Cioffi et al. 2012; Poltronieri et al. 2014).
